# A High-content screen identifies compounds promoting the neuronal differentiation and the midbrain dopamine neuron specification of human neural progenitor cells

**DOI:** 10.1038/srep16237

**Published:** 2015-11-06

**Authors:** Ji heon Rhim, Xiangjian Luo, Xiaoyun Xu, Dongbing Gao, Tieling Zhou, Fuhai Li, Lidong Qin, Ping Wang, Xiaofeng Xia, Stephen T. C. Wong

**Affiliations:** 1 Chao Center for BRAIN, Department of Systems Medicine and Bioengineering, Houston Methodist Research Institute, 6670 Bertner Avenue, Houston, TX 77030; 2 Department of Nanomedicine, Houston Methodist Research Institute, 6670 Bertner Avenue, Houston, TX 77030; 3 Department of Pathology and Genomic Medicine, Houston Methodist Research Institute, Houston, TX 77030; 4 Weill Cornell Medical College, Cornell University, New York, NY 10065

## Abstract

Small molecule compounds promoting the neuronal differentiation of stem/progenitor cells are of pivotal importance to regenerative medicine. We carried out a high-content screen to systematically characterize known bioactive compounds, on their effects on the neuronal differentiation and the midbrain dopamine (mDA) neuron specification of neural progenitor cells (NPCs) derived from the ventral mesencephalon of human fetal brain. Among the promoting compounds three major pharmacological classes were identified including the statins, TGF-βRI inhibitors, and GSK-3 inhibitors. The function of each class was also shown to be distinct, either to promote both the neuronal differentiation and mDA neuron specification, or selectively the latter, or promote the former but suppress the latter. We then carried out initial investigation on the possible mechanisms underlying, and demonstrated their applications on NPCs derived from human pluripotent stem cells (PSCs). Our study revealed the potential of several small molecule compounds for use in the directed differentiation of human NPCs. The screening result also provided insight into the signaling network regulating the differentiation of human NPCs.

Stem/progenitor cells hold great promise for the treatment of various neurological diseases^1^. A key challenge to realize their potential is to identify small molecule compounds effectively inducing the neuronal differentiation and the further functional specification. Numerous studies have been carried out on the directed neuronal differentiation of stem/progenitor cells. Prevailing protocols are mostly designed based on the principles learned from developmental biology^2^, by mimicking the appearance order of morphogens to recapitulate the developmental process and achieve the lineage specification^3^,^4^,^5^. However, the complexity of mammalian embryo development has prevented the faithful *in vitro* recapitulation, resulting in low neuronal yield with the undesired cell types representing a clinical concern such as the formation of tumors^6^. Techniques to further improve the neuronal differentiation efficiency remain to be developed. Moreover, current methods typically rely on the use of human recombinant proteins which makes the cost prohibitively expensive and impractical for routine use. The identification of small molecule compounds that can improve the differentiation efficiency, and reduce the use of recombinant proteins thus is highly desired for the clinical application of stem/progenitor cells.

In order to identify efficient and cost-effective compounds, as well as gain insight into the possible mechanism underlying, we carried out a chemical genetic screen^7^,^8^ to systematically characterize a comprehensive library of known bioactive small molecules, for their effects on the neuronal differentiation as well as mDA neuron specification from human ventral mesencephalon derived NPCs. Our study revealed major pharmacological classes effectively increase the rates of neuronal and/or mDA neuronal differentiation of human NPCs, and provided insight into the signaling network governing the differentiation of NPCs.

## Results

### Bioactive compounds promoting the neuronal differentiation from human NPCs

There are thousands of small molecule compounds which are known to be bioactive and have been previously used in clinic or research. We first collected more than 5,000 such compounds (**Materials and Methods**) to systematically characterize their effects on human NPCs by high-content screen^9^ following the procedure illustrated in Supplementary Fig. 1. In the experiment we used the human NPC line ReNCell VM, which was derived from the ventral mesencephalon of human fetal brain^10^. The origin of the cells enabled us to carry out the chemical screens on both the neuronal differentiation and the further mDA neuron specification of human NPCs, which occurs spontaneously in ReNCell VM cells upon the removal of mitogens bFGF and EGF (Supplementary Fig. 2). The spontaneous differentiation occurs at a low but consistent rate^10^, providing an ideal model for assessing the compound effect.

**Figure 1:**
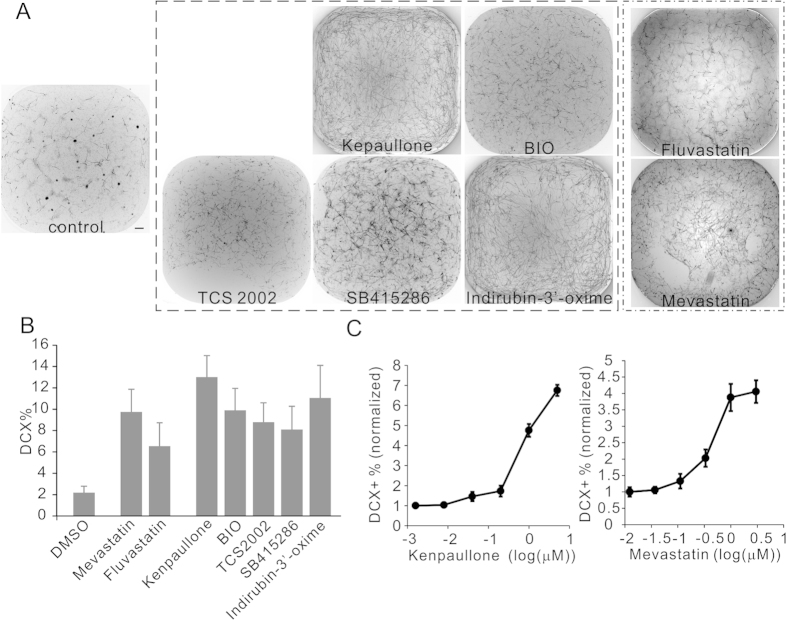
Major pharmacological classes promoting the neuronal differentiation of human NPCs. (**A**) The primary screen results of 5 GSK-3 inhibitors (dash line boxed) and 2 statin molecules (dash and dot line boxed), shown are the whole well images of 384-well plate stained with DCX antibody. (**B**) Quantification of the percentages of DCX+ cells (DCX+%) as the results of effective compounds treatment. The DCX+ % was calculated as the number of DCX+ cells divided by the number of total cells. Compounds were tested at 2 μM concentration. Controls received equal volume of DMSO treatment. (**C**) Dose responsive curves of the example compounds from the two pharmacological classes. Scale bar, 200 μm. The differentiation rates were normalized to the DCX+% in the DMSO control wells.

To start the neuronal differentiation screen undifferentiated ReNCell VM cells were seeded in laminin coated 384-well plates at a density of 1.8 × 10^4^ cells/cm^2^. Twenty-four hours later after the cells attached the medium was changed to remove the mitogens. Then to each well an individual compound from our collected library of bioactive compounds was added to a final concentration of 2 μM. Cells were differentiated in the presence of compound for 2 weeks with medium changed twice a week, before they were processed for immunoreactivity against Doublecortin (DCX), a widely used biomarker for early neuronal precursors to indicate the level of neuronal linage specification^11^. Each compound was tested 2 to 4 times and the result mean was used to select the primary hits. In this way over 5,000 compounds were scored on its promoting (positive scores from 1 to 3) or suppressing (negative scores from −1 to −3) effect on the neuronal differentiation from human NPCs based on the percentage of DCX+ cells generated in the differentiation (Supplementary Table 1). Further testing of the promoting compounds confirmed the effects of 21 compounds (Table 1), among which major effects were detected on two pharmacological classes, the GSK-3 inhibitors and the statins. For each class multiple chemicals were identified to significantly promote the percentage of DCX+ cells by more than 3 folds (Fig. 1A,B). Of these compounds kenpaullone and mevastatin stood out to be the most effective and therefore were used in the following up experiments (Fig. 1B). Titration experiment showed that they function in a dose-response manner with EC50 around 1 μM (Fig. 1C), suggesting their effects to be via specific pharmacological activities.

### Bioactive compounds promoting the mDA neuron specification from human NPCs

**Figure 2:**
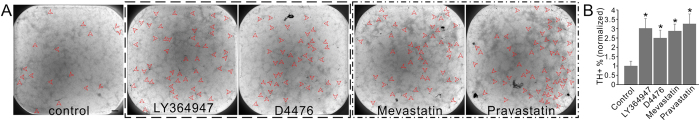
Major pharmacological classes promoting the dopaminergic neuronal differentiation of human NPCs. (**A**) The primary screen results of 2 TGF-βRI inhibitors (dash line boxed) and 2 statin molecules (dash and dot line boxed), shown are the whole well images of 384-well plate stained with TH antibody. The positive stained cells were pointed with red arrow heads for clarity as the image sizes are greatly reduced from the original. (**B**) Quantification of the cell differentiation rate in the validation experiments. The TH+ % was calculated as the number of TH+ cells divided by the number of total cells. The rate in the control wells treated with DMSO was normalized to 1. The compound concentrations are: LY364947 3 μM; D4476 2 μM; mevastatin 1 μM; pravastatin 1 μM. Controls received equal volume of DMSO treatment. *P < 0.05. Scale bar, 200 μm.

Similarly, high-content screen was carried out to test the compounds’ effects on promoting the mDA neuron specification of ReNCell VM cells, which is potentially valuable for the study of Parkinson’s disease^12^. Cells were grown in laminin coated 384-well plates at an initial density of 1.8 × 10^4^ cells/cm^2^, and differentiated in the presence of 2 μM compound for 4 weeks, then processed for immunoreactivity against tyrosine hydroxylase (TH), the most commonly used biomarker for mDA neurons^13^. Again each compound was tested 2 to 4 times and the result mean was used to select the primary hits. Over 5,000 compounds were scored based on their effect on the percentage of TH+ cells generated in the differentiation (Supplementary Table 2). As the spontaneous differentiation rate is low the compounds were only scored for their promoting effects (positive scores from 1 to 3). Re-test of the primary hits in 96-well plate format confirmed the effects of 30 compounds (Table 2), among which major effects were detected on two pharmacological classes, the TGF-βRI inhibitors and the statins. For each two chemicals were identified to significantly promote the number of TH+ cells generated from differentiation by more than 2 folds (Fig. 2A,B, high quality images are provided in Supplementary Fig. 3). Compounds LY364947 and mevastatin stood out to be the most effect of each pharmacological classes and were used in further experiments.

### Mechanisms of the major effective compound classes

In summary through the bioactive compound high-content screen approach we identified three major pharmacological classes, the GSK-3 inhibitors, statins and TGF-βRI inhibitors, to cause the significant enrichment of neuronal and/or mDA neuronal populations in ReNCell VM cell differentiation. The enrichment can possibly be attributed to the compounds’ promoting effects on signaling pathways driving the neuronal differentiation, or the proliferation/survival selection on the neuronal and non-neuronal cell types. To distinguish the mechanism experiments were carried out to test their effects on cell proliferation, apoptosis and lineage specification.

**Figure 3:**
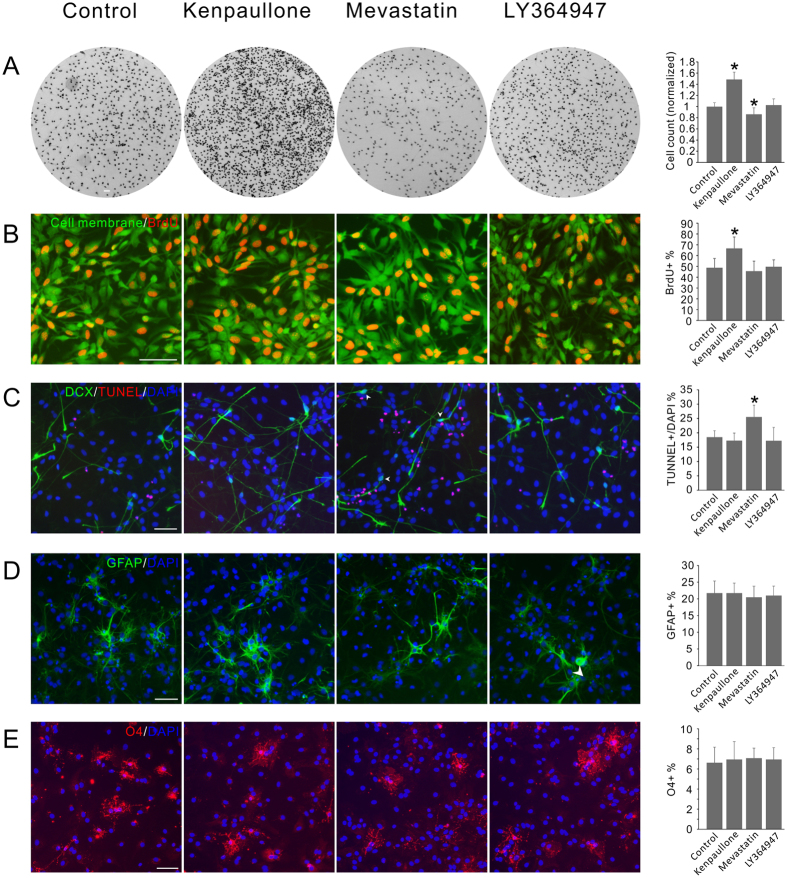
Effects of the Compounds on the cell proliferation, survival and glial differentiation. (**A**) Total cell count of the ReNCell VM cells after the treatment of various compounds. Kenpaullone significantly increased the cell number while mevastatin decreased it. (**B**) BrdU incorporation assay of the compound treated ReNCell VM cells. Kenpaullone significantly increased the number of proliferative cells while the other compounds had no effect. (**C**) Cell survival assay of the differentiated ReNCell VM cells treated with various compounds. Mevastatin significantly increase the number of apoptosis cells. Notably, apoptosis only occurred in undifferentiated cells, while not detected in the DCX+ neuronal cells (arrow head pointed). (**D**) Compound effect on the astrocyte differentiation of primary rat NPCs. None of the compounds significantly affected the differentiation rate. (**E**) Compound effect on the oligodendrocyte differentiation of primary rat NPCs. None of the compounds significantly affected the differentiation rate. In all the experiment the compound concentrations are: kenpaullone 3 μM; mevastatin 1 μM; LY364947 3 μM. Controls received equal volume of DMSO treatment. *P < 0.05. Scale bars, 50 μm.

During the screen we consistently observed changes of total cell numbers in the GSK-3 inhibitor and statin treated wells. To confirm the observation we seeded equal numbers of ReNCell VM cells in 96-well plates and incubated with individual compound selected from the three pharmacological classes, and counted the total cell numbers after differentiation for two weeks. While the TGF-βRI inhibitor LY364947 had no effect, the GSK-3 inhibitor kenpaullone significant increased the total cell number and the statin compound mevastatin decreased it (Fig. 3A). To distinguish whether the effects were achieved via changes in the number of proliferative cells or cell survival rate, we then carried out BrdU incorporation experiment. The result (Fig. 3B) indicated that kenpaullone treatment led to significantly increased number of proliferative cells, while mevastatin and LY364947 had no effect. As kenpaullone treatment caused the total cell number to increase by >30% (Fig. 3A), the increased population cannot be exclusively the neuronal cells which only constitute <15% of the total cells. Therefore we reasoned that the pro-proliferation effect of kenpaullone is not selectively on the neuronal population, and the possibility of kenpaullone to directly promote the neuronal differentiation pathways cannot be excluded.

Experiments were also carried out for cell survival measurement using the TUNEL assay, and the result showed that mevastatin elevated apoptosis rate while the other two compounds had no effect (Fig. 3C). Notably, apoptosis was exclusively detected in the non-neuronal cells, therefore the increased apoptosis level would contribute to the increase of the neuronal cell rate and at least partially accounted for the effect of mevastatin.

NPCs are tripotent cells which can differentiate into neurons, astrocytes and oligodendrocytes. In order to test whether the differentiation inducing capability of the compounds are selective to the neuronal lineage, experiments were carried to determine whether other lineages of cell differentiation were also altered by the compound treatment, using primary cultured NPCs isolated from the fetal rat ventral mesencephalon. The result showed that neither the astrocyte nor the oligodendrocyte differentiation was changed by the three compounds. Cells retained the differentiation capabilities and the glial cell differentiation rates remained the same as untreated cells (Fig. 3D,E). Taking together, our result indicated that the GSK-3 inhibitor increased both the expansion of total cells and the number of differentiated neurons, suggesting that the enrichment of neuronal cells is likely the result of increased neuronal differentiation rate, and a possible role of GSK-3 pathway in controlling the neuronal lineage specification. No effect on cell proliferation or survival was detected for the TGF-βRI inhibitors, so that their effect on mDA neuron enrichment is also a possible result of increased directed differentiation. While an effect on the cell differentiation capability cannot be excluded for the statins, at least part of their effect may be achieved through the selective survival of neuronal cells.

**Figure 4:**
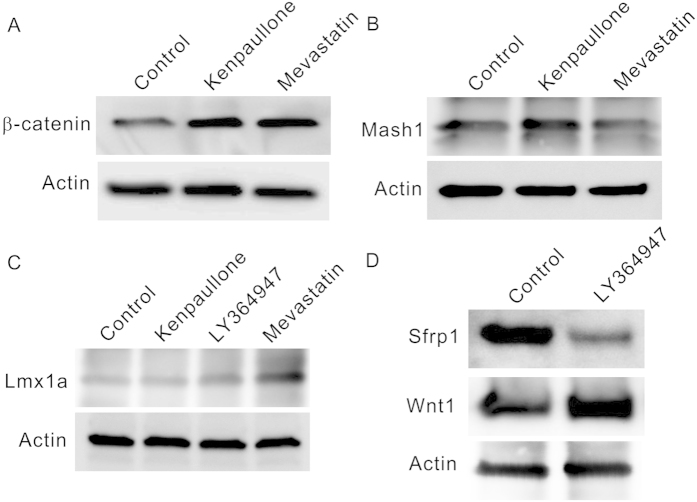
Compound effects on the signaling pathways controlling the NPC differentiation. (**A**) The effects of kenpaullone and mevastatin on Wnt/β-catenin signaling. Both compounds increase the amount of intact β-catenin. (**B**) The effects of kenpaullone and mevastatin on Mash1 expression. Kenpaullone increased Mash1 but not mevastatin. (**C**) The effects of kenpaullone, LY364947 and mevastatin on the Lmx1a expression. Increases of Lmx1a were detected for LY364947 and mevastatin. (**D**) The effect of LY364947 on Wnt signaling. LY364947 suppressed Sfrp1 and promoted Wnt1 expression. Full length blots are included in the Supplementary Information.

Next we tested how the known signaling pathways controlling NPC differentiation were affected by the compounds. Previously GSK-3 inhibitors^14^,^15^ and a different statin molecule^16^ have been reported to promote the neurogenesis of rodent NPCs. In these studies the enhancing of Wnt/β-catenin signaling was shown to account for the compound effects. We carried out western blot experiment and showed that the effect on Wnt/β-catenin signaling was replicated in human NPCs, with increased intact β-catenin level as a result of kenpaullone and mevastatin treatment (Fig. 4A). Moreover, the western blot result (Fig. 4B) also indicated an increase of Mash1^17^ expression by kenpaullone treatment, confirming a direct role of the GSK-3 inhibitors in promoting the neurogenesis pathway. Mash1 increase was not detected in mevastatin treated cells. However, treatment of mevastatin resulted in a significant increase of the expression of LMX1a, the intrinsic determinant of mDA neurons^18^. A weak LMX1a increase was also detected as a result of LY364947 treatment, consistent with their promoting effects on the mDA neuron specification of NPCs (Fig. 4C), suggesting the compounds’ effect to be achieved through direct activation of the differentiation pathway. LMX1a overexpression was not detected in the kenpaullone treated cells, consistent with the specific function on the neuronal differentiation but not the mDA neuron specification for the GSK-3 inhibitors (Fig. 4C). Recently TGF-β inhibition was shown to promote Wnt signaling, through the repression of the secreted frizzled related protein 1 (Sfrp1)^19^ which is a Wnt antagonist^20^. When this pathway was tested the result (Fig. 4D) indicated that this mechanism was also employed in the cultured ReNCell VM cells, leading to increased Wnt1 expression as a result of LY364947 treatment, to possibly account for the promotion of mDA neuron differentiation from NPCs.

### Functional differences of the major effective compounds and their combinatorial effects

**Figure 5:**
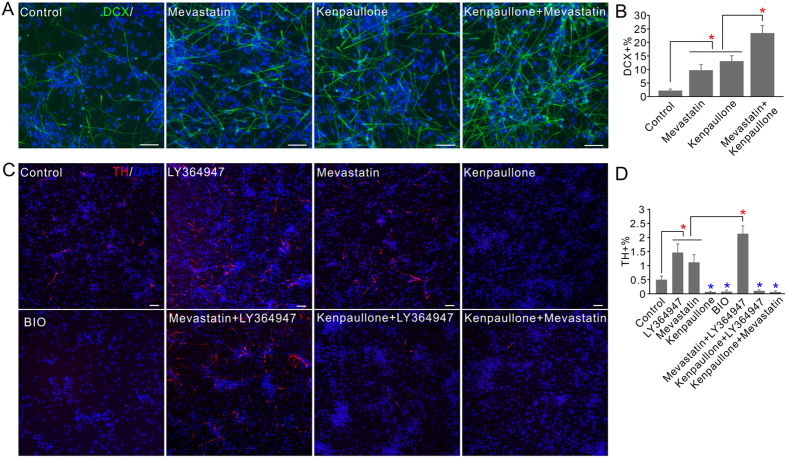
The effects of compound combinations on human NPC differentiation. (**A**) The effects of compound combinations on the neuronal differentiation of ReNCell VM cells. Cells were treated with 0.5 μm mevastatin; 3 μm kenpaullone; combination; or DMSO control during differentiation. Neurons were stained with DCX antibody. (**B**) Quantification of the percentage of DCX+ cells with or without treatment. (**C**) The effects of compound combinations on the dopaminergic neuronal differentiation of ReNCell VM cells. Cells were treated with 3 μM LY364947, 1 μM mevastatin, 3 μM kenpaullone, 1 μM BIO or the combinations of two compounds. Cells were stained with TH antibody. (**D**) Quantification of the percentage of TH+ cells after the treatments in (**C**). In all the experiment control received equal volumes of DMSO treatment. *P < 0.05 (red, significantly increased; blue, significantly decreased). Scale bars, 50 μm.

The western blot results indicated that there might be common mediators, e.g., β-catenin and LMX1a, for the promoting effects of the different bioactive compounds identified in our screen. However, the actions of different compound classes on NPCs are also distinct as indicated by their differential effects on the neuronal differentiation and mDA neuron specification, as well as on the cell proliferation and survival. Therefore through complementary mechanisms, their effects may be additive or synergistic. In both screens two major pharmacological classes were identified, we thus examined whether higher efficiency could be achieved through the combinations of them. Significant further enhancement was observed when the two neuronal differentiation promoting compounds kenpaullone and mevastatin were applied together (Fig. 5A,B). When the two dopaminergic differentiation promoting compounds, LY364947 and mevastatin were used in combination, significant enhancement were also observed (Fig. 5C,D).

The GSK-3 inhibitors were identified in the neuronal differentiation screen but not the mDA neuron specification screen, and increase in LMX1a expression was not detected in the GSK-3 inhibitor treated cells (Fig. 4C). We carried out experiments to examine their effect on the dopaminergic neuron differentiation, and the effect when used in combination with other promoting chemicals. Surprisingly, kenpaullone treatment led to the significant inhibition of the generation of TH+ dopaminergic neurons. Similar effect was also detected for a structurally different GSK-3 inhibitor BIO (Fig. 5C,D). Not only that, using of kenpaullone together with dopaminergic promoting compounds completely abolished their enhancing effects (Fig. 5C,D).

**Figure 6:**
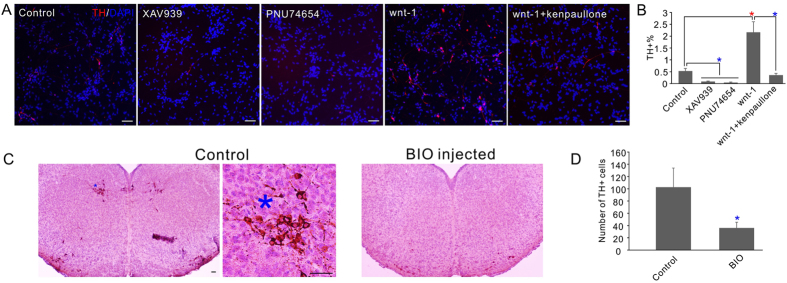
The effects of Wnt and GSK-3 inhibitor on mDA neuron differentiation. (**A**) The effects of wnt signaling on the dopaminergic neuronal differentiation of ReNCell VM cells. Cells were treated with 1 μM XAV939, 2 μM PNU74654, 20 ng/ml human recombinant Wnt-1 protein, 3 μm kenpaullone or the indicated combination. Cells were stained with TH antibody. (**B**) Quantification of the percentage of TH+ cells after the treatments in A. In all the experiment control received equal volumes of DMSO treatment. (**C**) Injection of cell permeable GSK-3 inhibitor BIO significantly inhibited the development of TH+ dopamine neuron in the developing mouse midbrain. Left, an example slice of the control animals; middle, high resolution image of the *region in left image to confirm the TH+ neuron morphology; right, an example slice of the BIO injected animals. (**D**) Quantification of the number of TH+ cells in the midbrain slices. Numbers shown are the averages of 9 slices from 3 different animals. Scale bars, 50 μm. *P < 0.05 (red, significantly increased; blue, significantly decreased).

Since GSK-3 inhibition activates Wnt/β-catenin signaling (Fig. 4A), the above result is seemingly contradictory to the known role of Wnt signaling on the mDA neuron development^21^,^22^,^23^. In order to confirm the eligibility of our *in vitro assay* and clarify the role of GSK-3 inhibition and Wnt signaling, we first directly tested the effect of Wnt-1 and showed that the recombinant protein significantly enhanced the number of TH+ cell differentiated from ReNCell VM cells (Fig. 6A,B). Consistently, treatment of the cells with Wnt inhibitors XAV939 and PNU74654 almost completely eliminated the TH+ cells (Fig. 6A,B), confirming the enhancing effect of Wnt on the development of dopaminergic neurons and the eligibility of our *in vitro* assay system. Interestingly, when the Wnt-1 recombinant protein is used together with kenpaullone, its enhancing effect was significantly inhibited (Fig. 6A,B). Moreover, injection of a brain permeable GSK-3 inhibitor BIO which also inhibited mDA neuron differentiation *in vitro* (Fig. 5C,D) significantly decreased the number of TH neurons *in vivo* in the developing mouse midbrain (Fig. 6C,D). Together these results confirmed the inhibitory effect of GSK-3 inhibitors on mDA neuron specification and suggested that the effect was achieved through pathways that are not related to Wnt signaling. In summary our results showed that the initial neuronal lineage differentiation and the later mDA neuron specification are individually regulated, and the same protein machinery may be utilized in these processes with distinct roles. These results also suggested that the three major compound classes identified in the screen act on NPCs differently, that may be selectively used individually or in combination to regulate the neuronal differentiation and mDA neuronal specification of human NPCs.

### Compound effects on human PSCs

**Figure 7:**
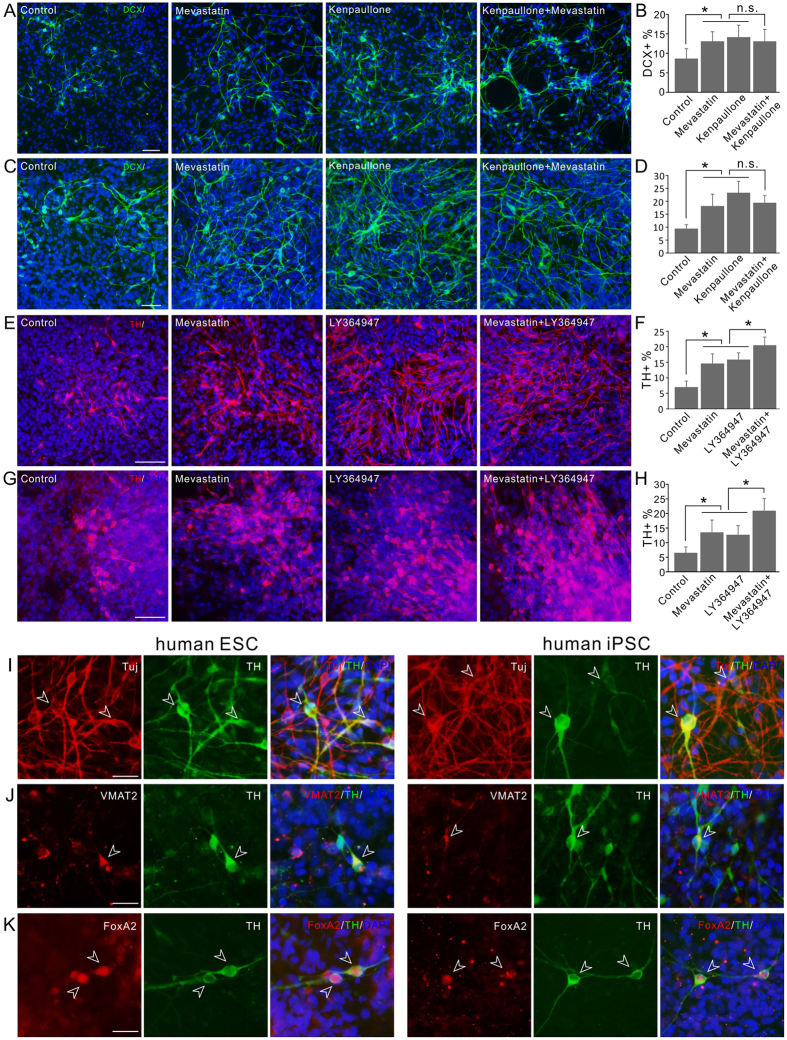
The effect of compounds on human PSC differentiation. (**A**) Images of DCX+ neuronal cells differentiated from human ESCs. Cells were treated with 0.5 μm mevastatin; 3 μm kenpaullone; combination; or DMSO control during differentiation. (**B**) Quantification of the percentage of human ESC differentiated DCX+ neuronal cells with or without treatment. (**C**) Images of DCX+ neuronal cells differentiated from human iPSCs. Cells were treated with 0.5 μM mevastatin; 3 μM kenpaullone; combination; or DMSO control during differentiation. (**D**) Quantification of the percentage of human iPSC differentiated DCX+ neuronal cells with or without treatment. (**E**) Images of TH+ dopaminergic neuronal cells differentiated from human ESCs. Cells were treated with 0.5 μm mevastatin; 3 μm LY364947; combination; or DMSO control during differentiation. (**F**) Quantification of the percentage of human ESC differentiated TH+ neuronal cells with or without treatment. (**G**) Images of TH+ dopaminergic neuronal cells differentiated from human iPSCs. Cells were treated with 0.5 μM mevastatin; 3 μM LY364947; combination; or DMSO control during differentiation. (**H**) Quantification of the percentage of human iPSC differentiated TH+ neuronal cells with or without treatment. (**I**–**K**) Characterization of the TH+ cells differentiated from mevastatin LY364947 combination treated human PSCs (left panels, human ESCs; right panels, human iPSCs). All the TH+ cells expressed neuronal marker Tuj (**I**), most of them also coexpressed VMAT2 (**J**). All the TH+ cells also expressed midbrain floor plate marker FoxA2 (**K**). Example cells were pointed with arrow heads. *P < 0.05. Scale bars, 50 μm in (**A**–**G**); 20 μm in (**I**–**K**).

Human PSCs, including embryonic stem cells (ESCs) and induced pluripotent stem cells (iPSCs), hold great promise as unlimited transplantation source material for treating neurological disorders^24^. To determine whether the bioactive compounds we identified can be applied to direct the differentiation of human PSCs, we tested their effects on the neuronal and mDA neuronal differentiation of human ESCs and iPSCs. Although to a less extent compared to the ReNCell VM cells, both kenpaullone and mevastatin were shown to significantly increase the neuronal differentiation of the NPCs derived from both human ESCs and iPSCs. (Fig. 7A–D). However, the combination of the two compounds did not lead to further increase of the neuronal differentiation rates (Fig. 7A–D), possibly due to the significantly increased toxicity especially affecting the differentiated DCX+ cells (Supplementary Fig. 4), which was not observed in the ReNCell VM cells. When the human PSCs were induced to differentiate, dopaminergic neurons were generated upon the patterning with FGF-8 and SHH. Both mevastatin and LY364947 were able to significantly increase the dopaminergic differentiation level, and their combination led to further increase of the number of TH+ dopaminergic neurons (Fig. 7E–H). The compound combination induced TH+ cells from PSCs exhibited typical neuronal cell morphology and were confirmed to express the classical neuronal marker βIII-tubulin (Tuj) (Fig. 7I). Many of the TH+ cells also expressed VMAT2, a critical transporter for the dopamine function of the monoamine neurons (Fig. 7J). Dopamine neurons can originate from several brain areas, of particular interest are the mDA neurons for their role in Parkinson’s Disease. FGF-8 treatment is able to direct the PSC differentiation to midbrain cells^25^, our result showed that mevastatin and LY364947 treatment did not interfere with the patterning and the resulting TH+ cells retained the expression of midbrain floor plate marker FoxA2^13^ (Fig. 7K). Together these results highlighted the potential of the compounds for the directed differentiation of human PSCs.

## Discussion

Previously studies have been carried out for identifying compounds inducing the neuronal differentiation from rodent SCs^14^,^26^,^27^, but the screening on NPCs derived from human brain tissue has not been reported. Here we reported a chemical screen on the NPCs originated from the ventral mesencephalon of human brain. It is noteworthy that our screen was carried out using small molecule compounds with known pharmacological activities, many of which are clinical used as drugs in USA or worldwide^28^, or widely used in biological research. Therefore the compounds identified in this study may help to improve the current protocol for the directed differentiation of human NPCs, to achieve the safe and cost-effective production of neuronal cells.

In both neuronal differentiation and mDA neuron specification screens statins were identified to be effective. Since consistent result was observed for several different statin members, including mevastatin, fluvastatin and pravastatin, the promoting effect is highly likely to be associated with the specific bioactivity of statins instead of an individual compound off-target. Statins are best known as HMG-CoA reductase inhibitors which inhibit the cholesterogenesis^29^. However, cholesterol inhibition is unlikely to directly promote the neuronal differentiation pathway, as cholesterol has been shown to be essential for the normal function of NPCs^30^. Cholesterol depletion caused massive defects in neural tube development^31^. Cholesterol was also known to be critical for the function of key NPC biomarker proteins such like prominin-1 (CD133), which directly interact with membrane cholesterol to form the microdomains in the apical plasma membrane^32^. Statins may have multiple pharmacological activities. Besides cholesterol control, statins were also known to improve the outcome following traumatic brain injury and stroke^33^,^34^ but the mechanism remains unclear. A recent study on simvastatin has associated this clinical effect with NPCs, and showed that simvastatin regulated isoprenoid synthesis, but not cholesterol, to enhance the Wnt signaling and adult neurogenesis^16^. The currently reported effect of statins on the neuronal differentiation of fetal NPCs is likely associated with the same pathway. And the result is consistent with our finding that GSK-3 inhibitors, which also enhance Wnt signaling by preventing β-catenin degradation, significantly promoted neurogenesis and were identified in the screen. However, statins (at least mevastatin) may also have additional biochemical function which further increases the neuronal cell rate when used in combination with the GSK-3 inhibitor kenpaullone. One likely explanation is that mevastatin contributed to reduce the number of undifferentiated cells as shown in Fig. 3C, possibly through cholesterol ablation. The result that statins also promoted the mDA neuron specification from NPCs was not reported before. The effect can be reasonably explained by its promotion on Wnt signaling.

Of the statin molecules identified in our screening, mevastatin showed the outstanding effects in both promoting the neuronal lineage differentiation and mDA neuron specification, on both the NPCs from human ventral mesencephalon and human PSCs. Therefore it may have potential in the directed differentiation of NPCs for the study of Parkinson’s Disease. In addition, differentiation inducing compounds may also find its application in cancer therapy, by targeting the brain tumors that are initiated by cancer stem cells^35^. Mevastatin is particularly of interest as it simultaneously induce the differentiation of stem cells and the apoptosis of undifferentiated cells, thereby help to eliminate brain tumor cancer stem cells. Our result also provided a possible explanation why long-term prediagnostic statin use may improve the survival following glioblastoma^36^.

The discovery of GSK-3 inhibitors to promote the neurogenesis is consistent with literature reports^14^,^37^. Recently kenpaullone was also identified through a compound screen to prolong the survival of stem cell derived motor neurons^38^. The effect of kenpaullone on motor neurons is not likely associated with its GSK-3 inhibition activity as other GSK-3 inhibitors failed to promote motor neuron survival. Instead, the inhibition of a different kinase, HPK1/GCK-like kinase (HGK), was suggested to be responsible for its effect motor neurons. In our study, at least five different GSK-3 inhibitors have been shown to enhance the neuronal differentiation efficiency. Therefore the effect is highly likely to be specifically through the GSK-3 inhibition, which then promote the Wnt signaling to enhance the neurogenesis.

Two findings in this study were surprising and seemingly contradictory to existing evidences. The first is the result that GSK-3 inhibitors promoted DCX+ neuronal differentiation but inhibited TH+ mDA neuron specification, as GSK-3 inhibition enhances Wnt signaling which is known to play a critical role in the neurogenesis in the midbrain floor plate^22^,^23^. Although the mechanism remains unclear, our result indicated that the effect was achieved through Wnt independent pathway(s). These results clarified the function of GSK-3 inhibition in mDA neuron development and added to the complexity of the role of GSK-3 in brain cell fate determination^39^. The other surprising result is the identification of TGF-βRI inhibitors to promote the dopaminergic neuronal differentiation, as TGF-β has been shown to be essential for the development of mDA neuron *in vitro* and *in vivo*^40^. In recent years, dual SMAD inhibition (TGF- β inhibitor combined with BMP inhibitor) protocol has been increasingly used to generate mDA neurons from human PSCs^41^. While the inhibitors was originally thought to enhance the generation of neural progenitors by inhibiting mesenchymal differentiation, a recent study revealed that TGF-β inhibition might up-regulate the SMAD-interacting protein 1 (S1P1), resulting in greater repression of Sfrp1 and the rise of Wnt1-Lmx1a levels, thereby directly promote mDA neuron differentiation^19^. Our result is consistent with this study, and provided more direct evidence that TGF- β inhibitor may promote the dopamine neuron differentiation from the embryonic midbrain NPCs.

## Methods

### NPC culture and differentiation

Human NPC line ReNCell VM was purchased from Millipore (Millipore, Billerica, MA, USA), which were derived from the ventral mesencephalon of human fetal brain and immortalized with the myc oncogene^10^. Primary NPCs were cultured from the ventral mesencephalon from the E14 rat embryos^42^. Tissues were dissected and dissociated with accutase to single cells, and grown in suspension to form neurospheres first. The spheres were then digested with Accutase (StemCell Technologies, Vancouver, Canada) to single cells and grown adherently as monolayer on laminin (Life Technologies, Grand Island, NY, USA) coated surface. The culture medium consists of knockout DMEM/F12 and StemPro neural supplement (Life Technologies, Grand Island, NY, USA) with 10 ng/ml bFGF and 10 ng/ml EGF (StemCell Technologies, Vancouver, Canada)^43^,^44^. To start the neuronal or glial differentiation, the cells were gradually switched to the differentiation medium consisting of Neurobasal and B27 supplement without bFGF or EGF, by changing half medium every two days for two weeks.

### Human PSC culture and differentiation

The H9 (WA09) human ESC derived NPCs was obtained from Life Technologies (Life Technologies, Grand Island, NY, USA)^45^. The human iPSC was purchased from ATCC (ATCC, Manassas, VA, USA). The iPSCs were cultured in feeder-free condition in Essential 8 medium (Life Technologies, Grand Island, NY, USA) with bFGF. Neural induction was started by culturing the cells in STEMdiff neural induction medium (StemCell Technologies, Vancouver, Canada) on Matrigel coated surface for 9 days. Cells were passaged in the same culture condition twice and then the derived NPCs were maintained in STEMdiff neural progenitor medium (StemCell Technologies, Vancouver, Canada) before they were used for the neuronal differentiation experiment. The neuronal differentiation was done following the same procedure as described above for the ReNCell VM and primary rat NPCs, in the absence or presence of indicated compounds.

The dopaminergic neuron differentiation was carried out following a procedure modified from published protocol^46^. Briefly, human PSC derived NPCs were first dissociated and grown in suspension to form neurospheres. The neurospheres were attached on laminin coated surface in neural differentiation medium consisting of DMEM/F12 and B27 (Life Technologies, Grand Island, NY, USA), and patterned with 50 ng/ml FGF-8 (Peprotech, Rocky Hill, NJ, USA) for 1 week followed by 100 ng/ml SHH (Peprotech, Rocky Hill, NJ, USA) and 50 ng/ml FGF-8 for 1 week, then SHH and FGF-8 were withdrawn to continue the dopamine neuron differentiation for another week. Cells were treated with indicated compounds starting from the patterning.

### Chemical Libraries

The compounds libraries used for this study included: the LOPAC library consisting of 1,280 pharmacologically active compounds (LOPAC, Sigma-Aldrich, St Louis, MO, USA); the Tocriscreen Mini library consisting of 1,120 biologically active compounds (Tocriscreen, Tocris Bioscience, Bristol, UK); the Spectrum Collection consisting of 2,320 compounds (Spectrum, MicroSource, Gaylordsville, CT, USA); the Prestwick Chemical library consisting of 1,200 compounds (Prestwick, Prestwick Chemical, Parc dinnovation, France); the Prestwick Phytochemicals Library consisting of 320 phytochemical compounds (Phytochemical, Prestwick Chemical, Parc dinnovation, France); the Prestwick Natural Compounds Library consisting of 240 natural compounds (Natural, Prestwick Chemical, Parc dinnovation, France).

### Compound screening

Undifferentiated ReNCell VM cells were seeded at a density of 1.8 × 10^4^ cells/cm^2^ in 384-well plate pre-coated with laminin. After 24 hours the medium were changed to remove the mitogens and each well was treated with a different compound at 2 μM concentration. Half of the medium was refreshed every two days with the compound level maintained at 2 μM. For neuronal differentiation screening cells were fixed after 2 weeks, and stained DCX antibody to determine the neurogenesis level. For dopaminergic neuronal differentiation screening, the medium were also supplemented with 10 ng/ml BDNF and 10 ng/ml GDNF, and the plates were fixed after 4 weeks of differentiation and stained with TH antibody to count the number of dopaminergic neurons. In both screenings, each compound was tested 2 to 4 times and the result averages were used to select the primary hits.

### Primary screening hits selection

Screening images were first subjected to a double blind manual analysis, in which each image was scored by an independent analyzer as 0 (absent of effect as untreated); or positive scores of 1 (minor enhancing effect), 2 (medium enhancing effect) or 3 (major enhancing effect); or negative scores of −1 (minor inhibiting effect), −2 (medium inhibiting effect) or –3 (major inhibiting effect). The results from all repeats and both analyzers were averaged and the compounds of scores ≥2 were selected to further test in 96-well plate format. Images acquired on the 96-well plates were quantified using the NeuriteIQ software^47^, to determine the percentages of neuronal differentiation (DCX+%, number of DCX positive cells/number of total cells), or dopaminergic neuronal differentiation (TH+%, number of TH positive cells/number of total cells). Statistical analysis was carried out to select the compounds with significant promoting effects (p < 0.05), which were designated as the primary screening hits and subjected to further analysis.

### Cell proliferation and survival assay

For the cell proliferation assay, ReNCell VM cells were seeded into laminin coated 96-well plates at a density of 4 × 10^3^ cells/well (1.25 × 10^4^ cells/cm^2^). Cells were treated with indicated compound or DMSO control for 48 hours before they are fixed and stained with Hoechst 33342 dye (Life Technologies, Grand Island, NY, USA). The plate was scanned with an ImageXpress Micro microscope (Molecular Devices, Sunnyvale, CA, USA) using a 4× objective to take an image covering the whole well for each well. Cell numbers were counted using the ImageJ software with an automatic nuclei counter plug-in. Cell proliferation was also measured by BrdU assay. Cells were grown in laminin coated 96-well plates, and treated with indicated compound for 48 hours. After that cells were pulsed with 10 μM BrdU for 2 hours, then stained with BrdU antibody (1:1000, Cell Signaling, Danvers, MA, USA). For the survival assay, cells were differentiated in the presence of indicated compounds. Then the cells were fixed, permeabilized and assayed with the Roche *in situ* Cell Death detection kit following the manufacturer’s instruction. Cells were also blocked and stained with additional markers as indicated.

### Immunostaining and western blots

The immunofluorescence staining of the cells were done with Alexa-fluor-488 or −568-conjugated secondary antibodies ((Life Technologies, Grand Island, NY, USA), and the cell nuclei were counterstained with DAPI. Antibodies against the following proteins were used at the following dilutions: DCX (1:500, Cell Signaling, Danvers, MA, USA), GFAP (1:100, Millipore, Billerica, MA, USA), O4 (1:100, R&D Systems, Minneapolis, MN, USA), TH (1:500, Cell Signaling, Danvers, MA, USA; 1:200, Millipore, Billerica, MA, USA). Fluorescence images were taken using an IX81 inverted microscope (Olympus, Tokyo, Japan). Image quantification was performed using the ImageJ software.

For western blot, cells were lysed in RIPA buffer (Fisher Scientific, Pittsburgh, PA, USA) in the presence of Xpert protease inhibitor cocktail and Xpert phosphatase inhibitor cocktail (GenDEPOT, Barker, TX, USA). Thirty microgram protein was loaded in each well and separated by 4–15% Mini-PROTEAN TGX precast gel (Bio-rad, Hercules, CA, USA) and transferred to a nitrocellulose membrane. The antibodies tested included β-catenin (1:1000, Cell Signaling, Danvers, MA, USA), Mash1 (1:1000, Abcam, Cambridge, MA, USA), Lmx1a (1:1000, Santa Cruz Biotechnology, Santa Cruz, CA, USA), Sfrp1(1:1000, Cell Signaling, Danvers, MA, USA), Wnt1 (1:500, Abcam, Cambridge, MA, USA), and actin (1:1000, Santa Cruz Biotechnology, Santa Cruz, CA, USA).

### Animal injection and brain slice immunohistochemistry

Animal experiments were carried out in accordance with the protocol approved by the Institutional Animal Care and Use Committee of Houston Methodist Research Institute. Groups of four C57BL/6 mice were injected intraperitoneally (i.p.) with 1 mg/kg brain permeable GSK-3 inhibitor BIO (Tocris Bioscience, Bristol, UK) or DMSO control daily for 4 days from E13 to E16. Forty-eight hours later the animals were sacrificed and brains were dissected and postfixed in 4% PFA overnight at 4 °C. Then the brains were dehydrated and embedded in paraffin. Sections were made from the midbrain at 10 μm thickness on a microtome. Slides from histologically comparable positions as confirmed by the H&E staining were used in the immunostainings. Immunohistochemistry was carried out with a Vectastain Elite ABC kit (Vector Laboratories, Burlingame, CA, USA) following the manufacturer’s manual. The rabbit anti-TH antibody was purchased from Millipore (1:200, Millipore, Billerica, MA, USA).

### Statistics

Statistical analyses were carried out using a two-tailed Student’s t-test. Data presented in the graphs are the mean values with the error bars representing the standard deviations (s.d.).

## Additional Information

**How to cite this article**: Rhim, J. *et al.* A High-content screen identifies compounds promoting the neuronal differentiation and the midbrain dopamine neuron specification of human neural progenitor cells. *Sci. Rep.* **5**, 16237; doi: 10.1038/srep16237 (2015).

## Supplementary Material

Supplementary Information

Supplementary Table 1

Supplementary Table 2

## Figures and Tables

**Table 1:** List of compounds promoting the neuronal differentiation of ReNCell VM cells.

Compound name	Compound description
4F 4PP oxalate	selective 5-HT2A antagonist
BIO	ATP-competitive GSK-3 inhibitor.
BWB70C	selective inhibitor of 5-lipoxygenase
Canrenone	aldosterone antagonist
DMPO	neuroprotective agent, radical spin trap
Ezetimibe	Inhibit cholesterol absorption in the small intestine
Flunixin meglumine	non-steroidal anti-inflammatory
Fluvastatin	HMG-CoA reductase inhibitor
Indirubin-3′-oxime	GSK-3 inhibitor; also inhibits other protein kinases
Isoproterenol hydrocloride	selective β-adrenoceptor agonist
Kenpaullone	GSK-3 inhibitor; CDK inhibitor
Mevastatin	HMG-CoA reductase inhibitor
n-methyl-metacryloyl-lupinine	quinolizidine alkaloid extracted from plant
Phenoxybenzamine hydrochloride	selective α-adrenoceptor blocking agent; calmodulin antagonist
SB 203580	selective inhibitor of p38 MAPK
SB 239063	potent, selective p38 MAPK inhibitor; orally active
SB 415286	competitive GSK-3 inhibitor.
Sotalol hydrochloride	β-adrenoceptor agonist
Spermine tetrahydrochloride	modulatory on the NMDA glutamate receptor
TCS 2002	GSK-3 inhibitor
Tolazoline hydrochloride	non-selective competitive α-adrenoceptor antagonist

**Table 2:** List of compounds promoting the dopaminergic neuronal differentiation of ReNCell VM cells.

Compound name	Compound description
1-Naphthyl PP1	Src family kinase inhibitor; also inhibits c-Abl
7-NINA	non-selective NOS inhibitor
AG 490	EGFR-kinase inhibitor; JAK2/3 inhibitor
Bifonazole	inhibit ergosterol synthesis; inhibit HMG-CoA
Cilostamide	selective inhibitor of PDE III
Clobenpropit dihydrobromide	highly potent H3 antagonist and H4 partial agonist
D 4476	CK1 inhibitor; TGF-βRI inhibitor
DAPT	γ-secretase inhibitor
HA14-1	Bcl-2 inhibitor
ICI 118,551 hydrochloride	highly selective β2-adrenoceptor antagonist
ICI-185,282	potent thromboxane receptor antagonist
Kynurenic acid	broad spectrum EAA antagonist
LY 364947	selective inhibitor of TGF-βRI
Mevastatin	HMG-CoA reductase inhibitor
MK-912	selective α2A-adrenoreceptor agonist
Naltrindole hydrochloride	non-peptide δ opioid antagonist
Nefazodone HCl	5-HT2, 5-HT1A receptor antagonist
ODQ	selective inhibitor of NO-sensitive guanylyl cyclase
Pranoprofen	non-steroidal anti-inflammatory
Pravastatin	inhibit HMG-CoA reductase
Prochlorperazine edisylate	D2 receptor antagonist
Purpurin	xanthin oxidase inhibitor
R(–)-Isoproterenol (+)-bitartrate	selective β-adrenoceptor agonist
R(+)-Terguride	dopamine receptor agonist.
SLV 320	potent and selective A1 antagonist
S-Sulfo-L-cysteine sodium salt	EAA receptor agonist
SU 5416	potent and selective VEGFR PTK inhibitor
Tyrphostin AG 112	protein tyrosine kinase inhibitor
Tyrphostin AG 494	protein tyrosine kinase inhibitor
Tyrphostin B44	EGFR-kinase inhibitor
