# Supplementary material for: A High-content screen identifies compounds promoting the neuronal differentiation and the midbrain dopamine neuron specification of human neural progenitor cells
Source: Sci Rep. 2015 Nov 6;5:16237. doi: 10.1038/srep16237 (PMC4635364; doi:10.1038/srep16237)
Supplement: Supplementary Information [file srep16237-s1.pdf]

A High-content screen identifies compounds promoting the neuronal differentiation and the midbrain dopamine neuron specification of human neural progenitor cells

Ji heon Rhim[1,†], Xiangjian Luo[1,†], Xiaoyun Xu[1], Dongbing Gao[1], Tieling Zhou[1], Fuhai Li[1,4], Lidong Qin[2,4], Ping Wang[3,4], Xiaofeng Xia[1,4,*], Stephen T. C. Wong[1,4]

[1]Chao Center for BRAIN, Department of Systems Medicine and Bioengineering, [2]Department of Nanomedicine, [3]Department of Pathology and Genomic Medicine, Houston Methodist Research Institute, Houston, TX 77030

[4]Weill Cornell Medical College, Cornell University, New York, NY 10065

[†]These authors contributed equally to this work

[*]Correspondence and requests for materials should be addressed to X.X. (xxia@houstonmethodist.org)

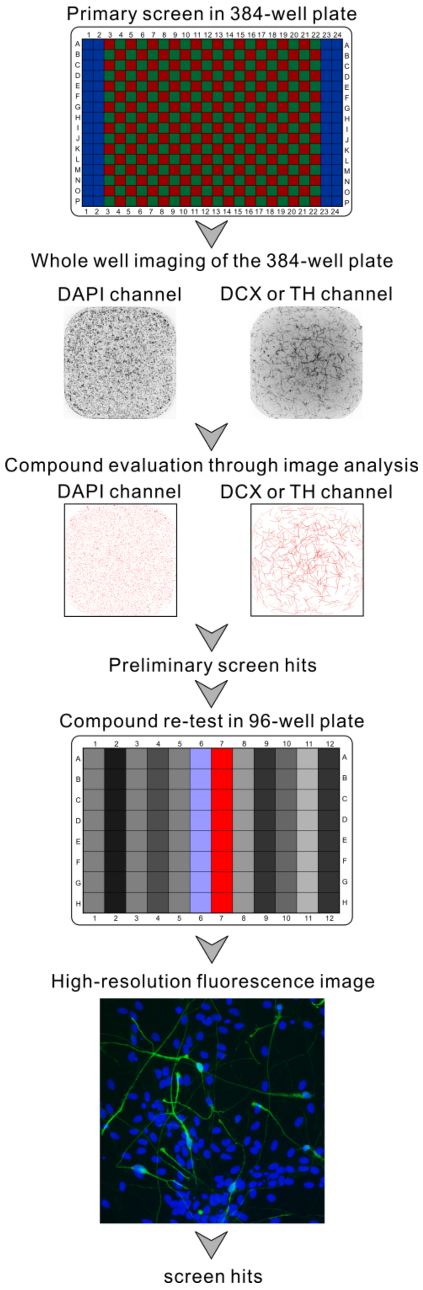

Supplementary Fig. 1 | High-content screen flow chart. Primary screen was carried out in 384-well plate with each compound tested in duplicate (Blue, control wells; red, duplicate #1; green, duplicate #2). After staining a low magnification image covering the whole well was taken for each well in two channels, to count the total cell number as well as the number of positive cells and evaluation the compound effect. Preliminary screen hits were re-tested in 96-well plate (Blue, DMSO control; red, positive control RA; grey, compound candidates) to confirm their effects and determine the final screen hits.

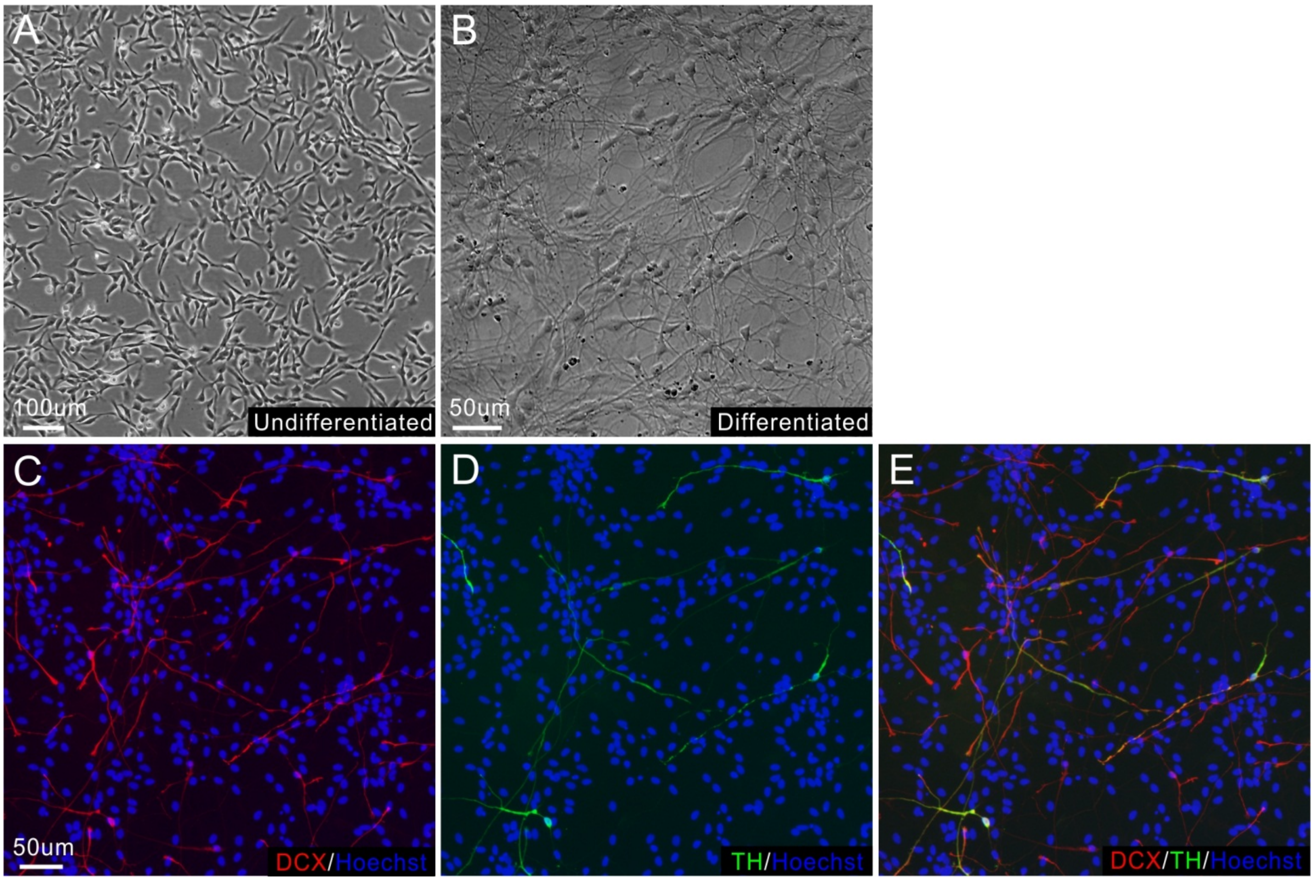

Supplementary Fig. 2 | Characterization of the ReNCell VM cells. (A) Cells can be propagated as undifferentiated NSCs in the presence of bFGF and EGF. Shown is a bright field image of the undifferentiated cells. (B) Neuronal differentiation occurs upon the withdrawal of bFGF and EGF. Shown is a bright field image of the differentiated cells. (C) Neuronal lineage specification can be identified by DCX staining during differentiation. (D) Dopaminergic patterning of the neurons can be identified by TH staining during differentiation. (E) Overlap of the DCX and TH stained images.

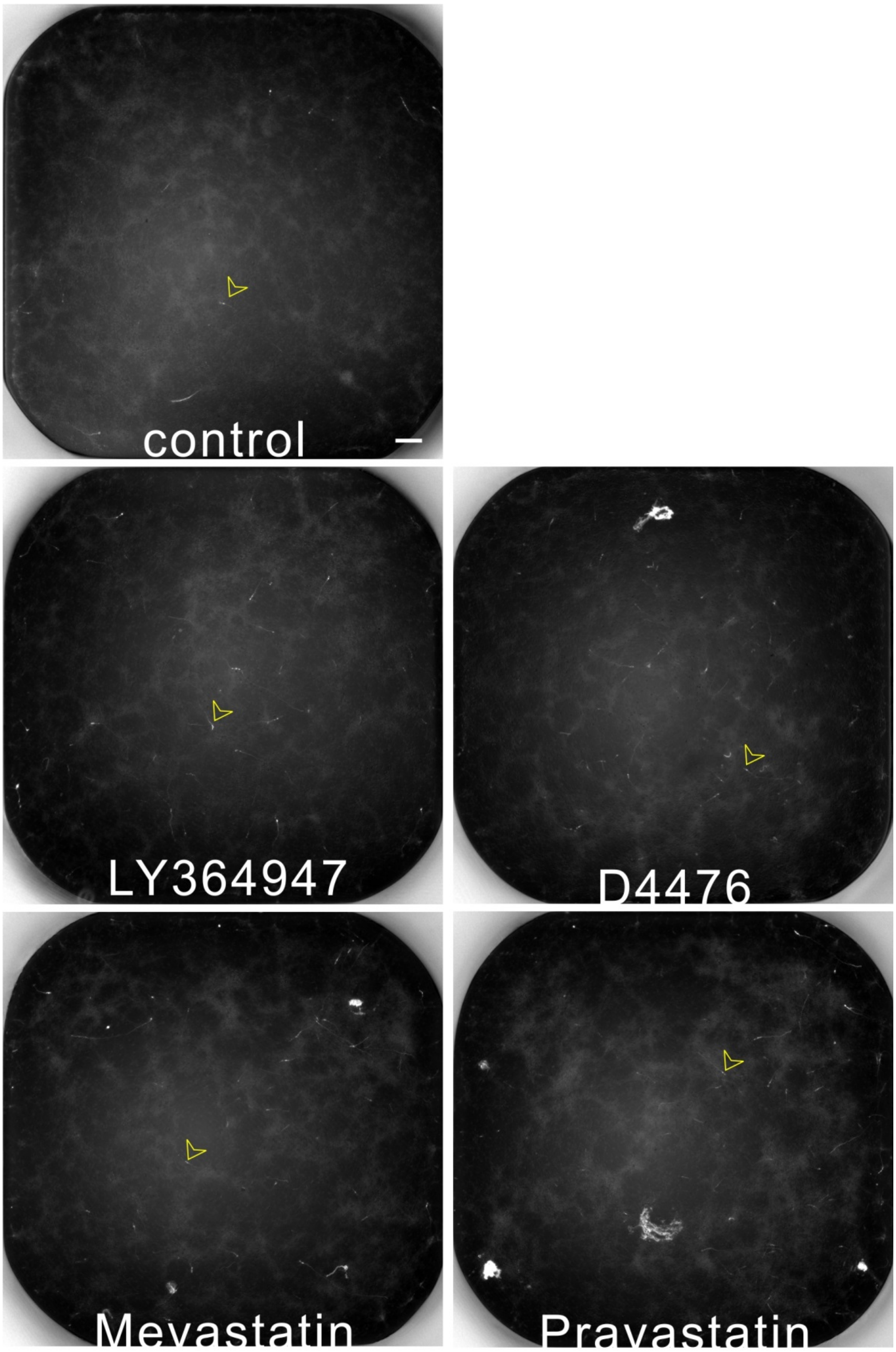

Supplementary Fig. 3 | High resolution images of the high-content dopaminergic neuronal differentiation screen. Arrow head pointed are examples of TH+ cells.

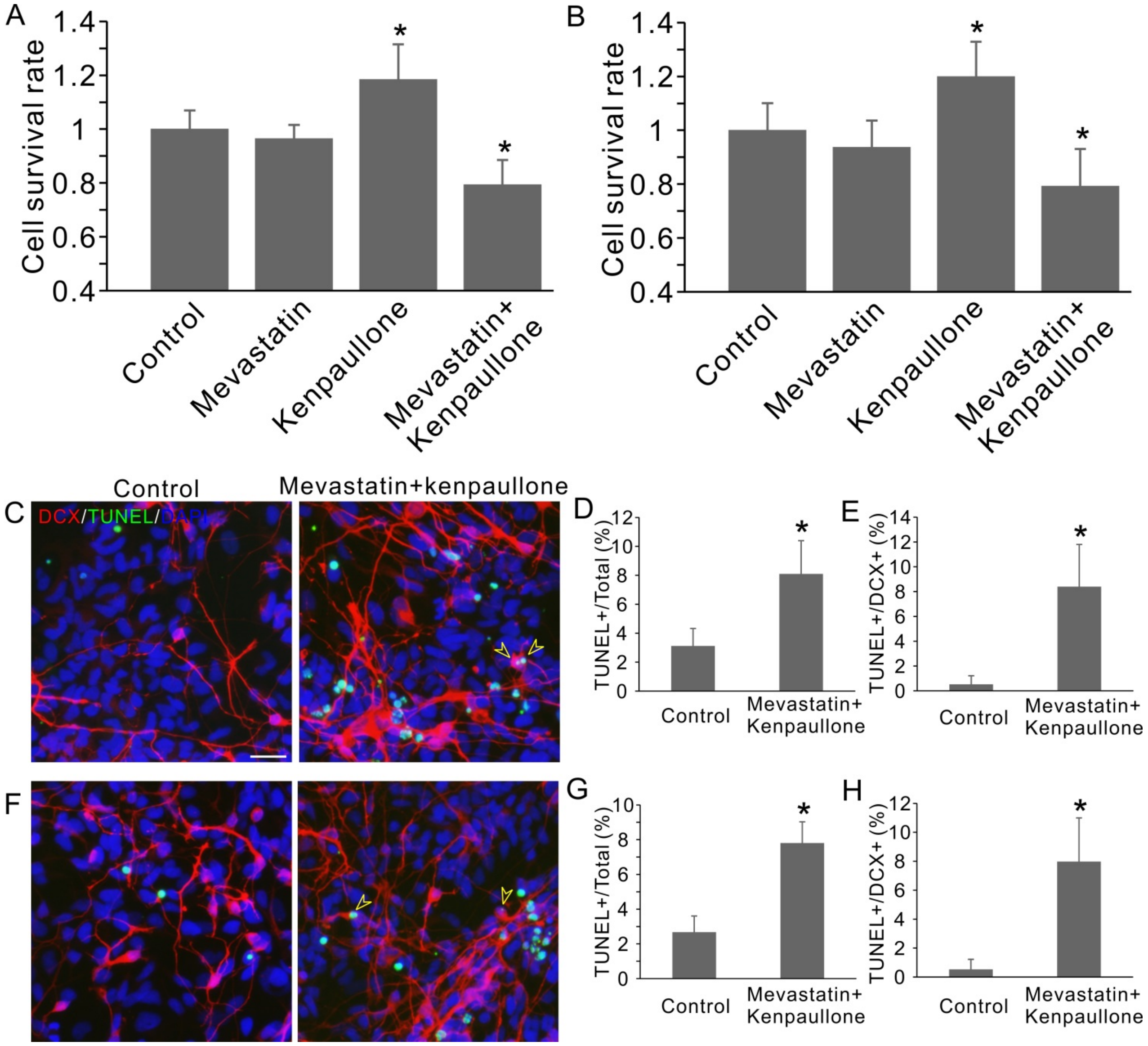

Supplementary Fig. 4 | The effect of mevastatin kenpaullone combination on the survival rate of human PSC differentiated cells. The combination of mevastatin and kenpaullone showed significant toxicity on the cells differentiated from both human ESCs (A) and iPSCs (B), leading to decreased number of survival cells. TUNEL staining confirmed that mevastatin and kenpaullone combination treatment significantly increased the apoptosis rate in the human ESC differentiated cells (C). Left, control; right, combination treated cells. Notably, apoptotic cells were rarely detected in DCX+ cells in the control experiment, while in the combination treated cells many DCX+ cells were stained with TUNEL (arrow heads). The quantification of the percentage of TUNEL+ in the total and DCX+ cells were shown in D and E. The same results were also observed in the human iPSC differentiated cells (F, G and H). *, $P<0.05$. Cells were treated with 3 μM kenpaullone, or 1 μM mevastatin, or both.

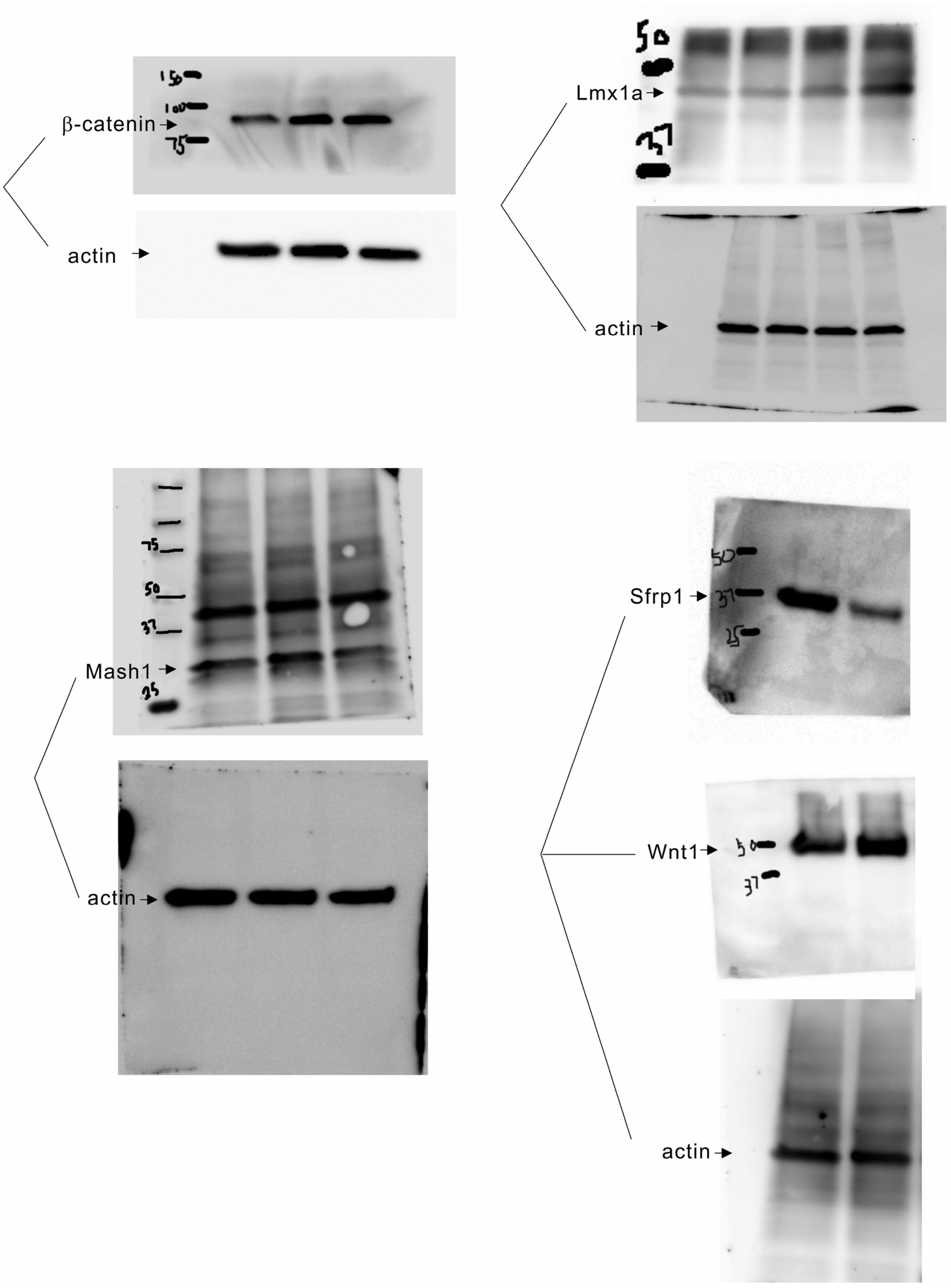

Full length blots for Figure 4.
